# The Role of Diagnostic Injections in Spinal Disorders: A Narrative Review

**DOI:** 10.3390/diagnostics11122311

**Published:** 2021-12-09

**Authors:** Brian Y. Kim, Tyler A. Concannon, Luis C. Barboza, Talal W. Khan

**Affiliations:** Department of Anesthesiology, University of Kansas Medical Center, 3901 Rainbow Boulevard, Kansas City, KS 66160, USA; tconcannon2@kumc.edu (T.A.C.); lbarboza@kumc.edu (L.C.B.); tkhan@kumc.edu (T.W.K.)

**Keywords:** diagnostics, injections, spinal disorders, facet arthropathy, lumbar radiculopathy, discogenic pain, discography, sacroiliac joint dysfunction

## Abstract

Neck and back pain is increasingly prevalent, and has increased exponentially in recent years. As more resources are dedicated to the diagnosis of pain conditions, it is increasingly important that the diagnostic techniques used are as precise and accurate as possible. Traditional diagnostic methods rely heavily upon patient history and physical examination to determine the most appropriate treatments and/or imaging studies. Though traditional means of diagnosis remain a necessity, in many cases, correlation with positive or negative responses to injections may further enhance diagnostic specificity, and improve outcomes by preventing unnecessary treatments or surgeries. This narrative review aims to present the most recent literature describing the diagnostic validity of precision injections, as well as their impact on surgical planning and outcomes. Diagnostic injections are discussed in terms of facet arthropathy, lumbar radiculopathy, discogenic pain and discography, and sacroiliac joint dysfunction. There is a growing body of evidence supporting the use of diagnostic local anesthetic injections or nerve blocks to aid in diagnosis. Spinal injections add valuable objective information that can potentially improve diagnostic precision, guide treatment strategies, and aid in patient selection for invasive surgical interventions.

## 1. Introduction

Neck and back pain is an increasingly prevalent condition in our aging population, and contributes significantly to global socioeconomic and healthcare burdens [1]. According to the Global Spine Care Initiative of 2015, low back and neck pain prevalence and disability has increased exponentially in the last 25 years, with over half a billion patients reporting symptoms of low back pain (LBP), and a third of a billion reporting neck pain lasting for at least 3 months [2]. Although patients are generally living longer thanks to advancements in modern medicine, morbidity and functional disability associated with age-associated spinal degeneration have also risen concomitantly, affecting an estimated 32–68% of the general population above age 65 [3]. With this increase in spinal pathology has come a concomitant increase in healthcare costs associated with advanced diagnostic imaging and treatments [4,5].

The economic burden related to back pain includes both direct and indirect costs. Direct cost variables include primary care visits, specialist visits, emergency room visits, hospitalizations, and physical therapy visits. A 2017 survey conducted in Spain consisting of 23,089 respondents estimated a cost of 2280.42 million euros related to direct care of LBP [6]. Indirect costs can be defined by loss of productivity, often represented in years lived with disability (YLD). According to the Global Burden of Disease study in 2015, LBP was the number one cause of global disability, responsible for 60.1 million YLD, which represents a 54% increase from 1990 [1]. As more resources are dedicated to the diagnosis of pain conditions, it is increasingly important that the diagnostic techniques used are as precise and accurate as possible.

Traditional diagnostic methods rely heavily upon patient history and physical examination to determine the most appropriate and cost-effective treatment and/or imaging studies. Though plain radiographs are utilized to rule out or detect bony abnormalities, such as fractures, advanced imaging techniques, such as magnetic resonance imaging (MRI) and computed tomography (CT), are often obtained to gain greater detail and further evaluate the surrounding neural structures and soft tissues. Although advanced imaging generally affords a high level of sensitivity for structural deformities, the diagnostic specificity and clinical correlation in degenerative conditions have been called into question [7,8,9]. Imaging sophistication and classification is undoubtedly improving [10], but there remains a lack of precision and positive predictive value when utilizing these traditional diagnostic methods. Imaging studies may highlight several structural abnormalities that may not correlate with pain. Alternatively, a patient may describe spinal pain without an evident source readily identifiable on imaging studies. For these reasons, physicians have sought additional methods for strengthening diagnostic accuracy.

There exists a wide variety of pain generators within the spine and surrounding structures. Identification of these pain generators is imperative in avoiding unnecessary treatments that may expose the patient to risks and cost without the intended benefit. Though traditional means of diagnosis remain a necessity, in many cases, correlation with positive or negative responses to injections may further enhance diagnostic specificity, and improve healthcare outcomes by preventing unnecessary treatments or surgeries. Here, we aim to review the most recent literature describing the diagnostic validity of precision injections, as well as their impact on surgical planning and outcomes.

## 2. Methods

A search was conducted in PubMed using a combination of the following relevant search terms and key phrases: diagnostic; predictive value; physical exam; injection; block; selective nerve root; transforaminal epidural; neuroforaminal stenosis; lateral recess stenosis; radiculopathy; facet arthropathy; facet joint; medial branch block; discography; discogenic pain; sacroiliitis; and sacroiliac dysfunction. Article titles and abstracts were screened for relevance. Reference lists of selected articles were searched for additional relevant literature for inclusion. Results are summarized and presented narratively below.

## 3. Facet Arthropathy

The zygapophyseal or facet joints are the bilateral contact points between individual spinal vertebrae that allow the body to bend, twist, flex, and extend, while also providing stability by preventing excessive movements. Over time, these joints begin to degenerate, and may become significant sources of pain. Facet arthropathy can present in any spinal segment excluding the sacrum, but is most commonly found in the lumbar spinal segments, followed by the cervical spine. Patients with facet arthropathy classically present with chief complaints of non-radiating back or neck pain worsened by axial extension and rotation or prolonged standing. Though undoubtedly a necessary component of evaluation, patient history and physical examination lack robust diagnostic precision.

Studies have historically demonstrated inconsistent validity and reliability when using history and physical examination alone for the diagnosis of facet arthropathy [6,7]. For example, a collection of physical exam findings known as Revel’s criteria, including intersegmental instability, tenderness to palpation over the facet joint, and exacerbation with standing flexion, extension, and/or rotation, had initially shown significant predictive value, but ultimately could not be validated in later studies [11,12]. In a 2018 meta-analysis of seven studies (*n* = 463), Usunier et al. found that intersegmental instability tests along with mechanical palpation have a relatively high diagnostic specificity (0.91 and 0.88, respectively) and sensitivity (0.74 and 0.61, respectively). However, the authors admit these findings were highly dependent on individual clinician experience and skill, impairing generalizability [13]. In an even larger systematic review performed by Maas et al., 129 different combinations of patient history and physical examination were evaluated, but failed to find conclusive evidence to support their diagnostic accuracy in facet arthropathy, demonstrating the importance of additional diagnostic techniques [14].

The initial surgical evaluation of back pain almost always includes diagnostic imaging, such as plain films, CT, and/or MRI. When facet arthrosis is detected, the Weishaupt scale is commonly used to grade degenerative severity (0 to 3 scale) based on the degree of facet joint narrowing, sclerosis, hypertrophy, and osteophyte formation [15]. Although this scale has been used as a means of surgical planning by identifying specific levels of pathology and disease severity, validity studies have reported conflicting results at best [16,17,18]. One retrospective observational study by Park et al. included 165 patients, and demonstrated an association between facet angle difference (facet tropism) at L3–4 and disc height at L5–S1 to positive medial branch block response; however, the study’s power is limited and, thus, requires further investigation [19].

In the absence of a consistent diagnostic gold standard, paravertebral facet joint injections and medial branch nerve blocks (MBB) with local anesthetic infiltration have emerged as potential tools for facet arthropathy diagnosis and treatment. Each facet joint has two nerve root contributions originating from above and below the joint (e.g., facet arthropathy at level L4–5 requires blockade of the L3 and L4 root medial branches). Thus, if the facet joint capsule itself is not targeted, then blockade of both medial branches must be performed to fully anesthetize the target joint. These blocks are traditionally performed under fluoroscopic guidance, but recent studies have proven ultrasound-guided techniques to be equally safe and efficacious in both cervical and lumbar regions [20,21,22].

The ultrasound-guided technique for blockade of the L5–S1 medial branch on the right is as described: initially place the ultrasound probe in the longitudinal axis along the midline lumbar spine, and identify the hyper echoic L5 spinous process and the median S1 crest. The L4–5 and L5–S1 facet joints and S1 dorsal foramen can be found by sliding the transducer approximately 1–2 cm laterally. The L4 and L5 transverse processes can be found by sliding approximately 1 cm further. After marking these landmarks, the probe is then rotated to a transverse orientation, and directed over the S1 superior articulating process (SAP), with the L5 transverse process identified laterally. Using an in-plane technique, a needle is advanced at 45 degrees to the skin in a lateral to medial direction until bony contact is made at the junction of the SAP and L5 transverse process. Aspiration is performed to rule out intravascular needle placement, and local anesthetic is deposited. The implementation of this ultrasound-guided technique may greatly improve accessibility by decreasing time and cost associated with the procedure, while also eliminating radiation exposure to both the patient and providers. However, these techniques are new and require further investigation before widespread implementation can be recommended.

The response to these particular diagnostic injections is often used as predictors of outcome after nerve rhizotomy or radiofrequency ablation (RFA). In 2018, Cohen et al. demonstrated in the Facet Treatment Study that, when compared with a placebo control, both the medial branch block and facet joint injection provide significantly better prognostic value before radiofrequency nerve ablation [23]. The block to radiofrequency technique, however, has proven to have several uncontrolled factors that may limit generalizability of existing studies, including local anesthetic volume, number of sequential diagnostic blocks, interpretation of block success (improvement percentage), and RFA needle orientation, thus, creating a wide range of reported sensitivities and specificities. For example, a cadaveric study by Wahezi et al. demonstrated that small differences in cervical and lumbar MBB injectate volume had significant implications in study results [24,25]. When 0.5 mL and 0.25 mL were compared, both resulted in reliable blockade of the medial branches, but dorsal spread to the superficial muscles and distal nerves was significantly increased in the 0.5 mL cohort, blocking additional potential pain generators, and diminishing its specificity. Because of the non-standardized nature of the MBB technique, and conflicting results in existing data, systematic reviews are required to make any conclusions.

In a large comprehensive systematic review, Schneider et al. examined several similar technical limitations of the diagnostic MBB [26]. First, the single block technique was found to have an unacceptably high false positive rate of 38–45%. The addition of the “dual comparative block”, or second diagnostic block utilizing local anesthetic with differing duration of action, greatly increased the likelihood of capturing true positives. For example, one study demonstrated a robust 88% specificity, but a diminished sensitivity of 54% when analgesic duration was considered in this comparative block [27]. Second, injection into the facet joints themselves adds no more predictive value than sham treatments. Third, the threshold for a positive block response is still largely variable within the literature, with 50–100% analgesic relief set as the standard for positive response. Diagnostic accuracy does increase with increasing thresholds for positive response, but sensitivity suffers in turn. Fourth, a larger RFA needle gauge (16–18 g vs. 20–22 g) and placement in parallel fashion was found to be more effective than the perpendicular orientation in relation to the nerve. Finally, the pooled data showed that the group utilizing the single injection technique had a 57% chance of obtaining greater than 50% relief at six months, whereas the double block technique group had a 58% chance of obtaining greater than 50% relief, a 36% chance of obtaining 80% relief, and a 23% chance of obtaining 100% relief at six months, suggesting similar rates of success, but a much greater degree of relief in the double block group. Furthermore, the most rigorous study examined showed a 56% chance of 100% relief at six months when complete relief was reported with two diagnostic MBBs [28].

## 4. Differentiating Lumbar Radiculopathy

Neural foraminal stenosis causing nerve root compression is one of the most common causes of low back pain and disability in the United States, with one study reporting a 73% prevalence in patients presenting with low back and leg pain [29]. Compression of dorsal nerve roots in the neural foraminal space can be caused by multiple deformities, including facet joint hypertrophy, osteophyte projections, disc herniation, spondylolisthesis, or, most commonly, disc degeneration. Surgical interventions for lumbar and cervical radiculopathy entail some form of mechanical decompression of the injured nerves. The determination of specific interventions, however, depends on the pathophysiology of disease. Discectomies or laminotomies may be performed for symptomatic herniated discs, whereas fusion may be performed when degenerative disc disease or spondylolisthesis is the cause of neural irritation and radicular symptoms. Successful spinal surgical outcomes rely heavily upon precise diagnosis of affected vertebral levels.

Pain distribution patterns, motor testing, and provocative physical tests allow for identification of laterality and even specific levels of injury. Diagnostic imaging then serves to further elucidate underlying pathology, and offers grading scales to assess severity of disease. A retrospective study performed by Tecer et al. included 57 symptomatic patient MRIs, and found that high intensity zones (HIZ) on T2 imaging, indicative of annular tears, had significant correlation with the response to transforaminal epidural steroid injection (TFESI) at week 2, and patients with visible nerve root impingement had significant improvement at 3 months post-treatment [30]. Ekedahl et al. also found high-grade subarticular nerve compression on MRI was associated with significant one-year improvement in visual analog scale (VAS) and Oswestry Disability Index (ODI) scores after TFESI [31]. However, definitive correlation between history and physical examination/imaging findings and presence of disease at specific vertebral levels remains to be seen [32,33,34]. Thus, history, physical examination, and diagnostic imaging have significant limitations, even as they remain the cornerstones of the initial evaluation of patients with radicular pain. Additional diagnostic testing is, therefore, necessary to increase specificity before committing to invasive and costly surgical interventions.

Despite its widespread use, the diagnostic accuracy of selective nerve root blocks (SNRB) depends on several factors, including variable technique, composition/volume of injectate, and needle position. Due to these variables, the reported accuracy of the test has ranged widely from 31–100% [35]. In a prospective study comparing 1 vs. 4 mL of contrast injectate, Kang et al. demonstrated an intraforaminal spreading pattern to adjacent levels in 24.5% of the 4 mL injectate group vs. 0% in the 1 mL injectate group, thereby suggesting smaller injectate volumes to enhance precision [36]. In a more recent study, however, Makkar et al. demonstrated increasing specificity with increasingly less injectate volumes: 78.8% selectivity after injecting 0.2 mL of dye; 33.3% after injecting 0.6 mL; and 6% after injecting 1.2 mL [37]. Another prospective study found that lateral intervertebral foramen needle tip position also improves specificity, demonstrating a lower incidence of epidural spread to adjacent nerve roots [38]. Despite its technical variables, SNRB has a growing body of evidence that supports its use in the surgical planning phase.

SNRB has been closely studied in the surgical literature as well. In a randomized controlled trial evaluating 99 patients undergoing selective endoscopic microdiscectomy, Li et al. reported significantly improved VAS pain scores and ODI scores, and higher rates of surgical success (89.6% vs. 68.6% excellent/good response via McNab clinical outcome scoring) in patients diagnosed via SNRB immediately prior to microdiscectomy [39]. Yue et al. found similar results when evaluating 101 patients undergoing surgical decompression, reporting good outcomes in 91% of patients with a positive SNRB vs. 60% with a negative SNRB [40]. Finally, in a large, 9-year retrospective study including 1839 patients, Lewandrowski et al. found that selective nerve blocks offer high predictive diagnostic value for improved clinical outcome after lumbar endoscopic transforaminal decompression, with a calculated 90.17% sensitivity, 70.79% specificity, and 98.38% positive predictive value [41]. Although positive findings have been reported, it is important to note that the consensus data remains mixed and inconclusive. In a systematic review comparing 341 patients across six studies evaluating patient selection for lumbar decompression surgery using SNRBs, Beynon et al. found a pooled sensitivity of 90.9% [83.1 to 95.3] and specificity of 22.0% [7.4 to 49.9] when using successful surgical outcome as the reference [42].

Although SNRBs have proven diagnostic and therapeutic value, there are several procedural risks and complications involved, including drug reactions, epidural bleeding or infection, nerve damage, and post-dural puncture headache. One literature review reported a 2.4–9.6% incidence of minor complications, including a temporary increase in pain, dural puncture headache, or vasovagal reactions [43]. Major complications include any event causing permanent neurologic injury, including spinal abscess, spinal cord infarct, or epidural hematoma. These are exceedingly rare, with evidence reported in case reports and closed claims databases. From 2009 to 2013, 16 patients experienced spinal cord infarcts, nine patients experienced epidural hematoma, and nine patients experienced soft-tissue infection [44]. Furthermore, particular awareness of critical adjacent vascular structures, such as the vertebral arteries and great radicular arteries, should be noted in the cervical and lumbar transforaminal approaches, respectively. Contrast use is therefore recommended to rule out intravascular uptake. Although arterial injury and spasm has been theorized as a potential ischemia-inducing mechanism, intravascular injection is also feared, especially when particulate steroids are used, since severe neurologic complications, including stroke or paralysis, have been linked to its use. Because of these risks, the use of ultrasound guidance with real-time tissue and needle tip visualization has emerged as a potentially advantageous technique. Jang et al. describes the ultrasound-guided cervical SNRB technique at the C7 nerve root as follows [45]. First, the ultrasound probe is placed in a transverse orientation, and C5–7 nerve roots are identified, using the C7 transverse process as a reference point. The optimal image of the nerve root, radicular artery, and other surrounding vessels are obtained using probe manipulation and color Doppler. Then, the 25-gauge needle is inserted using an in-plane technique in the posterior to anterior direction until the needle tip lies dorsal to the C7 nerve root. The injectate solution is inserted after confirmation of extravascular placement via negative pressure aspiration. Recent studies validate this ultrasound-guided technique as an effective alternative in both the cervical and lumbar spine, which may reduce not only procedural time and radiation exposure, but, more importantly, intravascular puncture incidence [45,46,47,48]. Although many studies support its non-inferiority, intravascular infiltration cannot be ruled out in the sonographic view. Two studies comparing the rate of vascular uptake in fluoroscopic vs. ultrasound-guided cervical transforaminal epidural steroid injections found no difference between the two techniques [49,50]. A narrative review by Ehsanian et al. therefore suggests a combination of initial ultrasound guidance with fluoroscopic confirmation to minimize the adverse effects and shortcomings of either singular technique [51]. The optimal technique has yet to be elucidated, and currently requires additional adequately powered superiority studies.

## 5. Discography

Discography uses image guidance to direct an injection of contrast material into the center of one or more spinal discs to help identify the source of back pain. Provocative discography is a highly specific test in which pressurization of a disc is performed in order to elicit a painful response and, thus, identify the source of the patient’s back pain. It can also be used to help guide the treatment of abnormal intervertebral discs. A non-painful discogram usually rules out the need for surgical intervention. Throughout the years, the use and diagnostic value of discography has been called into question, as non-invasive imaging techniques have been developed to help identify disc pathologies. One such imaging technique would include the use of the MRI T2 HIZ for the identification of abnormal disc morphology. A 2017 meta-analysis suggested a strong correlation between the HIZ on a T2-weighted MRI and positive outcomes of discography [52]. Developed by the International Association for the Study of Pain (IASP)/Spine Intervention Society (SIS), clear guidelines currently exist to ensure appropriate execution of provocative discography without potentially negative long-term side effects. These include determining the appropriate level of pain response (greater than 8/10), volume of contrast injected, and level of disc pressurization, among other criteria. When adhering to these standards for appropriate technique and data interpretation, provocative discography is associated with a very low false positive rate. This was demonstrated by a meta-analysis that found a low false positive rate of 9.3% per patient and 6.0% per disc when applying the SIS/IASP technique and criteria [53].

Issues with utilizing imaging alone to guide surgical correction arise when anatomical disruption does not always correlate with symptomatic pain, and, likewise, when the lack of disruption does not equate to lack of pain. This is common in patients with back pain, and may lead to overtreatment or undertreatment, significantly obscuring which patients may benefit from surgical intervention. It can be argued that imaging alone is not sufficient for identifying a pathogenic intervertebral disc [54].

With regards to the use of discography and its purpose for surgical planning, it has been found that discography has a large impact on patient selection. Xi et al. demonstrated that the use of provocative discography in conjunction with CT scanning provided a useful way to appropriately and accurately identify patients who would be good lumbar fusion surgical candidates, specifically patients who had concordant discogenic pain with annular tears on discography and CT, respectively [54]. Of the 43 patients with a mean age of 48.8 presenting with refractory low back pain, 18 qualified for and underwent interbody arthrodesis, with levels ranging from L3 to S1. The 18 patients who underwent the procedure met certain criteria, including concordant pain on discography with annular tears on CT, annular tears present at one or two contiguous intervertebral levels, and at least one negative control disc (negative discography/CT). Patient outcomes were evaluated using post-procedure VAS and ODI scales, as well as SF-36 (mental and physical quality of life components), with a median follow time of 18 months. Most notably, VAS and ODI scores significantly improved from preoperative baseline at 2 weeks, 6 months, and ≥12 months post-surgery in patients selected using discography with CT scan. Xi and team specifically state that provocative discography with subsequent CT scanning tended to provide a basis for the justification of lumbar fusion surgery in patients with low back pain of annular damage etiology. Ultimately, this study concludes there is an association between discogenic pain and annular tears as determined by provocative discography with post-procedure CT scanning. It is worth noting this study delineates advantages of obtaining a CT scan as opposed to fluoroscopy alone, as CT imaging provides a superior resolution to clearly visualize dye leakage. Further, these patients experience symptomatic and functional improvement when managed surgically [54].

An extensive 2018 systematic review suggested stronger evidence exists for the use of discography as a diagnostic tool for the identification of lumbar discogenic pain. However, evidence was weaker for the identification of cervical discogenic pain, and almost non-existent for thoracic discogenic pain. This is due to significant internal inconsistency in the lumbar spine, and extremely high internal inconsistency in the cervical discography [55].

Although the literature comparing ultrasound-guided vs. fluoroscopy-guided discography is limited, a 2019 case study outlines clear and reasonable advantages of ultrasound-guided discography over fluoroscopy guidance. These include better visualization of soft tissue, such as blood vessels and neural structures, shorter procedure times, and decreased radiation exposure to both the proceduralist and patient [56].

There has been growing concern regarding the safety of provocative discography. Studies have suggested this procedure may cause serious adverse events, such as chronic back pain and acceleration of disc degeneration, although evidence is conflicting [57,58]. A 2015 prospective 10-year matched cohort study has shown a correlation between provocative discography and disc surgery events, serious low back pain events, disability events, and medical visits [57], although it is not without its limitations. For instance, study subjects were selected from a pool of patients at greater risk for disc degeneration than the overall population; thus, results may not be applicable to all patients undergoing the procedure. Attrition is another important limitation of this study, as 40 of 150 patients were lost to follow-up. A 2012 in vitro study using cultured human annulus cells found that radiocontrast exposure may be a contributing factor to reduced proliferation, increased cell death, and/or apoptosis after adjustments for osmolality [58]. This study certainly has some weaknesses, for example, the three-dimensional in vitro environments may provide higher local concentrations to cells at the injection site compared to in vivo, which may factor into the study’s outcome. Although more studies need to be performed to assess possible long-term complications of provocative discography, current evidence shows that long-term risk of disc degeneration, disc disruption, and inferior clinical outcomes have all been associated with over pressurization of the disc during discography rather than provocative discography performed according to IASP/SIS standards [53]. Finally, a narrative review by Migliore et al. describes different intradiscal injection techniques, including guidance by fluoroscopy, CT, and CT plus fluoroscopy. Comparison of various efficacy and safety outcomes found that results were not easily comparable due to differences in highly variable procedural techniques, study designs, and a limited number of studies, thus, warranting more research on the efficacy and safety of these procedures [59].

Although provocative discography may be regarded by some to be an outdated procedure, it still serves a prominent diagnostic role in discogenic back pain. This is especially true when structural abnormalities are present, with studies supporting its accuracy in diagnosing lumbar pathologies. Discography has also been shown to be a useful tool for surgical planning purposes. When used in conjunction with CT scanning, discography has been shown to appropriately identify lumbar fusion surgical candidates confirmed by evaluating outcomes. Although there is a lack of robust literature comparing ultrasound vs. fluoroscopy-guided discography, multiple advantages of ultrasound utilization are clear. When conforming to clearly specified standards and guidelines, discography proves to be a safe and useful procedure for the appropriate identification of discogenic type pain, and the selection of appropriate surgical candidates.

## 6. Sacroiliac Joint Dysfunction

The sacroiliac (SI) joint is a complex anatomical structure in its function, innervation, and composition. The SI joint connects the spine to the pelvis, functioning in the movement of the lower extremities, and playing a large role in the transmission of forces. It is composed of both synovial and cartilaginous portions, with the inferior portion being synovial, and the superior functioning closer to a cartilaginous joint. The primary role of the joint is to aid in stability. Joint motion occurs in multiple planes simultaneously, including flexion/extension, rotation, and translation. Innervation of the joint is highly variable, even differing from one side to the next on the same patient [60]. Fortin et al. established innervation of the posterior joint and ligaments primarily originates at the S1–S3 dorsal rami, with a contribution from L5 [61]. Although the innervation may be complex, it is well established that SI joint pain can result from both intra- and extra-articular nociceptive stimuli resulting from inflammation and instability.

Though low back pain is an incredibly well-documented pathology plaguing today’s population, it is postulated that SI joint pain is drastically underdiagnosed. One review estimated that the SI joint contributed to 10–38% of all cases of low back pain [60]. The typical presentation of pain originating from the SI joint includes pain below the belt line with radiation to the lower extremity and groin. It is noted that radiation of pain below the knee is rare [62]. With such a large prevalence of SI joint pathology contributing to lower back pain, this underdiagnosis may be a result of difficult diagnostic measures.

Current practice utilizes a combination of history, provocation maneuvers, imaging, and diagnostic blockade to aid in the diagnosis of SI joint pain. Provocative SI joint maneuvers include the distraction test, compression test, Gaenslen test, thigh thrust test, sacral thrust test, and flexion, abduction, and external rotation (FABER) test. Three positive provocative maneuvers are classically required to diagnose sacroiliitis. Telli et al. evaluated the validity and reliability of provocation tests in the diagnosis of SI joint dysfunction in 156 patients [63]. When evaluated individually, the FABER test demonstrated the highest sensitivity at 91.4%, and the Gaenslen resulted in the lowest at 56.4%. Radiography, CT, and MRI may all provide some insight into the underlying pathology of the SI joint; however, they have demonstrated very low sensitivity as a solitary diagnostic modality [64]. Radiographic images of the joint are difficult to obtain and profile the joint well. CT of the SI joint demonstrated a sensitivity of 57.5%, and specificity of 69% in patients with SI joint-mediated pain [65]. MRI is the most sensitive imaging technique for identifying sacroiliitis; however, it is still limited in identification of other SI joint pathologies [60]. The International Association for the Study of Pain (IASP) proposed criteria for the diagnosis of SI joint dysfunction, which included three positive provocative maneuvers, and relief with local anesthetic injection into the SI joint or the lateral branch nerves [66].

The fluoroscopy-guided block is the gold standard for diagnostic or therapeutic purposes when treating SI joint pain [65]. Generally, a 75% reduction of pain following a diagnostic SI joint block is considered positive. If 50–75% of the pain is reduced, the SI joint may be considered a major contributor to pain of the lower back. For patients that did well with surgical fusion, the response to an intra-articular local anesthetic injection providing 75% relief or greater and lasting at least 30–60 min in duration was consistent. A study by Polly et al. demonstrated a good response to SI joint fusion in patients with 50% relief from diagnostic SI joint block, suggesting that those patients with a 50–74% reduction in pain may find further intervention, such as SI joint fusion, beneficial [67]. A randomized prospective trial evaluated lateral branch pulsed RFA vs. intra-articular steroid injections, and found that pulsed RFA of the dorsal rami and the S1–3 lateral branch nerves provided superior and durable relief with functional improvement [68].

As studies have shown, SI joint pain is an underdiagnosed source of chronic low back pain. Though the diagnosis of SI joint pathology may be complex, a multimodal approach, including a thorough history, physical exam provocation maneuvers, imaging, and fluoroscopic-guided diagnostic blockade, provides a much more deliberate and focused approach to treating SI joint pain.

## 7. Conclusions

The traditional approach to evaluating a patient with back and/or leg pain begins with the patient history, physical examination, and thoughtful diagnostic imaging. Despite our best efforts, the etiology of pain may be difficult to ascertain via these traditional methods. There is a growing body of evidence supporting the use of diagnostic local anesthetic injections or nerve blocks to aid in diagnosis. Significant, but rare, complications exist for each procedure, including bleeding, infection, and ischemia secondary to particulate embolism or vasospasm. Advanced techniques utilizing ultrasound or CT image guidance in conjunction with fluoroscopy may reduce complications, but these techniques are relatively new, and validation in large comparison studies is still needed.

A recent systematic review by Sebaaly et al. reported a failed back surgery syndrome incidence of 10–40% in laminectomy patients, with significant causation attributed to poor patient selection and incorrect level of intervention [69]. This highlights the importance of an accurate diagnosis before exposing the patient to definitive surgeries with potential long-term ramifications. Spinal injections add valuable objective information that can help guide treatment strategies, and, furthermore, aid in patient selection when considering risky and invasive surgical interventions. Several studies discussed above depict a positive predictive trend for discography, selective nerve root blocks, and sacroiliac joint blocks, but again, robust, high-quality, and adequately powered randomized controlled studies are needed. In conclusion, there exists immense potential to improve diagnostic precision and surgical selection in intractable back pain patients. With the objective data derived from diagnostic injections, and close collaboration between the specialties of interventional pain and spine surgery, better outcomes for patients are certainly possible.

## Data Availability

No new data were created or analyzed in this study. Data sharing is not applicable to this article.
